# Durability of Thermal Renders with Lightweight and Thermal Insulating Aggregates: Regranulated Expanded Cork, Silica Aerogel and Expanded Polystyrene

**DOI:** 10.3390/gels7020035

**Published:** 2021-03-25

**Authors:** André Morgado, António Soares, Inês Flores-Colen, Maria do Rosário Veiga, Maria Glória Gomes

**Affiliations:** 1 CERIS, Instituto Superior Técnico, Universidade de Lisboa, Av. Rovisco Pais, 1049-001 Lisboa, Portugal; andresantosmorgado@gmail.com (A.M.); ortiz.soares@gmail.com (A.S.); ines.flores.colen@tecnico.ulisboa.pt (I.F.-C.); 2 Laboratório Nacional de Engenharia Civil, Av. Brasil 101, 1700-066 Lisboa, Portugal; rveiga@lnec.pt

**Keywords:** accelerated aging cycles, freeze/thaw cycles, regranulated expanded cork, silica aerogel, expanded polystyrene, thermal render, lightweight aggregate

## Abstract

Following the trend of energy-efficient construction, renders with thermal insulation properties have been studied for replacing conventional renders. However, there are still few studies on the durability of these renders that may become a barrier for their implementation. In this study, the performance of lightweight renders for thermal insulation to accelerated aging cycles and freeze/thaw cycles is discussed. For this purpose, renders with regranulated expanded cork (GEC), silica aerogel (SA), and expanded polystyrene (EPS) were produced and tested for compressive strength, ultra-sound velocity, Young’s modulus, and thermal conductivity before and after accelerated aging cycles (hygrothermal, IR and freeze/thaw cycles). With this study, a comparison between the influence of different aggregates on renders is carried out in order to understand their effect on different properties of renders.

## 1. Introduction

External renders are critical in the construction as they are the first barrier to degradation agents and play an important role in water-tightness, thermal insulation, masonry, and structure protection by exerting a decisive influence on their durability [1,2]. However, over time, renders suffer a gradual reduction in their performance in terms of their protective characteristics due to climate agents of degradation [2,3,4,5,6].

Also, due to the high volumes of natural resources consumed, society is increasingly concerned about sustainability and construction methodologies. Thus, it is urgent to adopt constructive solutions that aim to increase energy efficiency, sustainability and, consequently, its durability [5,7,8]. Moreover, durable solutions in buildings contribute directly to a reduction in the amount of waste produced, air pollutant gases and a more sustainable construction. With this concern, the study of durability is an increasingly important issue in the construction industry, being intrinsically related to the building quality, climatic conditions and the user’s quality of life [7,9,10].

For assessing the durability of new solutions, it is essential to identify the relevant degradation agents such as temperature, solar radiation, rain, humidity, wind, among others, since diverse materials react differently to the agents to which they are subjected [10,11,12,13]. For the evaluation of aging effects, building materials are usually subjected to long term natural outdoor climate exposure or accelerated laboratory aging tests in order to estimate their potential long-term service ability under environmental conditions of use [10,12,13,14,15].

Following the trend of energy-efficient construction, renders with thermal insulation properties have been developed and applied as an alternative to conventional renders in order to reduce heat transfer in buildings [16,17,18,19]. To achieve thermal renders, lightweight aggregates are added in order to minimize the thermal conductivity and the thermal bridging effect in structural elements, that results in reducing both the heat losses and the effects of condensations [16]. Two of the most used lightweight aggregates for thermal renders are expanded polystyrene (EPS) and cork granulate (CG) [20].

Cork is a renewable resource, extracted from bark of *Quercus suber* L. without damaging the tree [21], quoted by [22], is composed by closed cells and its main constituent is suberin which gives its hydrophobicity. Cork is a material with density between 100 kg/m^3^ and 140 kg/m^3^, and a low thermal conductivity between 0.035 and 0.070 W/m.K. One of the by-products of cork used in construction materials is the regranulated expanded cork (GEC) [23,24,25], which results from the pulverization of the rejected (uneven) upper and lower parts of the blocks or defective panels or those obtained from demolitions [26,27,28].

Expanded polystyrene (EPS) is produced by pre-expanding polystyrene beads, resulting in a thermoplastic polymer, rigid and closed-cell material with good thermal insulation properties (0.032–0.040 W/m.K) [16,29,30,31]. The use of EPS can be justified by its ultra-lightweight (with density less than 300 kg/m^3^) non-absorbent properties and is using as a petroleum by-product [32,33].

A cutting-edge material that has recently been associated with high performance thermal renders is silica aerogel (SA). This material has very low thermal conductivity (0.012 to 0.021 W/m.K) [34,35] that results from the combination of low density (between 3 and 500 kg/m^3^), high porosity (above 90%) and small mesopores (typically between 4 and 20 nm) [36,37,38]. Thus, although silica aerogel is not yet widely used in renders, this is an emerging material in building thermal insulation solutions and results from their durability and reliable long-term performance are crucial for their widespread implementation [13,39,40,41,42,43].

Information on the durability of silica aerogel and renders incorporating silica aerogel is even scarcer. Ihara et al. [44] observed for silica aerogel that wetting cycles have a negative impact of up to 10% on thermal conductivity even if the silica aerogel surface is hydrophobic. The same authors claim that once the hydrophobic surface, that usually consists of methyl groups, may decompose under solar irradiation over time, it may be necessary to determine the aerogel’s aging performance in terms of the solar irradiation. However, those authors study focusses only silica aerogel as isolated material, so when introducing it into a render or plaster mixture, the behaviour of the set (binder/aggregate) may be different.

In fact, when aggregates are incorporated into renders, it is necessary to take other factors into account. For example, cork has a good durability as an isolated material [28] although, according to Barnat-Hunek et al. [19,23], render with expanded cork (20 vol% of total mixture) as the only aggregate is inappropriate as a durable construction render due to the high mass loss after frost resistance. On the other hand, renders with EPS show a high loss in compressive strength after freeze-thaw cycles, due to micro cracks by the expansive effect of ice [45].

Regarding aerogel-based materials, Berardi & Nosrati [13] observed that high levels of relative humidity had the greatest impact (variations between 6% and 10%) on the thermal performance of aerogel-based plaster with 70–90% of aerogel and strong UV radiation alternated to high moisture and temperature levels, resulted in greater increase in thermal conductivity (15% and 17%) than high humidity alone (12% and 16%) for blaster with 50% and 25% of aerogel. However, since these variations where smaller than for pure plaster (22% for humidity alone and 25% for strong UV radiation alternated to high moisture and temperature levels), these authors concluded that, despite the influence of humidity and UV radiation, the performance of the aerogel-enhanced materials after aging is still very close to their original condition. Gomes et al. [46] also achieved a huge increase of thermal conductivity (up to four times mores) of aerogel-based renders for moisture contents, calculated by volume, greater than 0.25 m^3^/m^3^.

From the several studies reviewed on this subject [12,19,23,45,47,48], it is possible to understand that the specific heat capacity of aggregates, pore structure and the interconnection between aggregates and binder can have an important role in thermal renders durability.

There are already several studies that address the properties and performance of renders with incorporation of thermal insulating aggregates, from physical to mechanical properties [16,19,23,43,49,50,51,52,53,54,55]. However, there is still a lack of knowledge on the durability of these renders that may become a barrier for the implementation of these thermal renders such as silica aerogel-based render [44].

This study discusses the durability of renders with thermal insulation aggregates (silica aerogel, regranulated expanded cork, and expanded polystyrene). The compositions of the tested renders were deliberately designed not to focus on the impact of the varying percentages of aggregates, because it is usually subject of analysis [7,16]. In this case renders with 100% of each aggregate in a volumetric ratio 1:4 (binder:aggregate) were tested under accelerated aging tests (hygrothermal, infrared (IR) and freeze/taw cycles) to discuss thermal and mechanical evaluation.

## 2. Results and Discussion

The consistence of renders at fresh state (Table 1) are within the ranges provided from EN 1015-2 [56] for the corresponding bulk density of renders at fresh state. Thus, it was assumed that the renders have a consistence at fresh state suitable for its use at hardened state and for the evaluation of the behaviour under accelerated aging.

Reaching the hardened state, almost all renders had bulk density values close to those obtained at fresh state. Exception made for aerogel-based render (C(SA)) which achieved a considerable reduction (169 kg/m^3^). The values of the dry bulk density of the renders are summarized in Table 2.

### 2.1. Freeze/Thaw Accelerated Aging

To evaluate the effects of freeze/thaw, the specimens were tested for compressive strength, ultra-sound velocity, Young’s modulus and thermal conductivity before and after freeze/thaw procedure, with the results summarized in Table 3.

**Figure 1:**
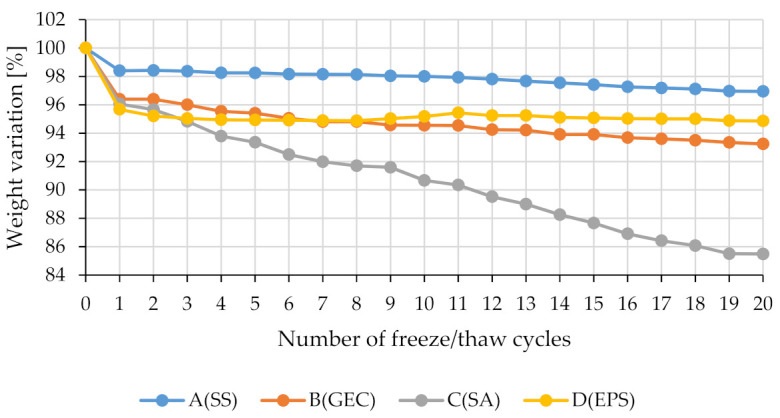
Weight variation along the freeze/thaw cycles.

To understand when to stop the testing, the renders specimens were weighed after each cycle. Since no renders reached a mass variation of more than 30% after 20 cycles, the freeze/thaw procedure was completed for all renders after 20 cycles, as shown in Figure 1 with the development of weight variation at each cycle.

**Figure 2:**
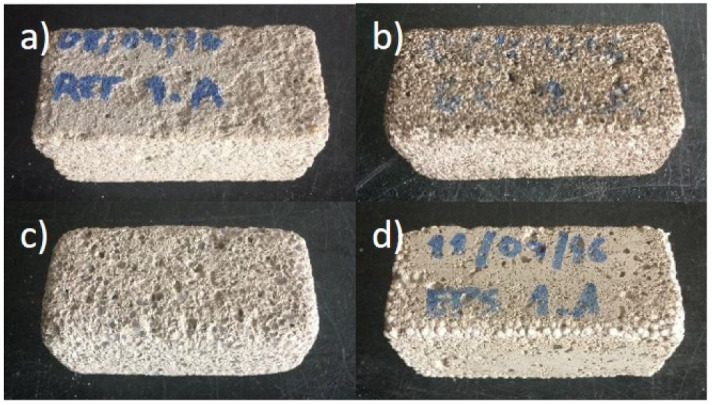
Half standard specimens after freeze/thaw cycles: (**a**) A(SS); (**b**) B(GEC); (**c**) C(SA); (**d**) D(EPS).

When evaluating the weight variation with the freeze/thaw cycles, it can be seen in the Figure 1 that, for the majority of the renders under study, the greatest weight loss occurs in the first cycle, which may indicate some superficial disintegration of material, maintaining a stable rate in subsequent cycles. This behaviour is shown in Figure 2, where for renders A(SS), B(GEC) and D(EPS) it is still possible to see part of the intact surface after the 20 freeze/thaw cycles, indicating that for these renders the number of cycles had a smaller effect in weight variation. Only aerogel-based render (C(SA)) demonstrates a significant reduction in weight at each cycle, which justifies the higher final variation compared to the other renders. This render achieves a weight loss of 14.52% after 20 cycles, a very high value considering the maximum variation of 7% (after 300 cycles) proposed as acceptable for concrete [57].

In Figure 2c, one can also observe craters (empty spaces) where the aerogel grains would be in render C(SA), indicating that there was a detachment of this with the freeze/thaw cycles that can be explained by the dosage of surfactants used to promote bond between aerogel granules and the remaining matrix. Since different types of aerogel have different needs of surfactants to achieve a good bond between aerogel and the remaining mixture [51,58], after analysing the detachment of aerogel with the freeze/thaw cycles, it is assumed that the dosage of surfactants in the studied formulation may not have been the most appropriate. Thus, further studies are needed for different aerogel formulations, subject not in the scope of this paper.

Render D(EPS) is the one that surface shows the lowest degradation (Figure 2d), showing only a slight degradation on the edges of the specimen. This render, along with render B(GEC), as in Figure 2b, showed the smallest variation in weight among renders with lightweight aggregates. Control render A(SS) presented the highest weight loss in absolute terms (Table 3). However, since this is a high-density render, its weight percentage variation was reduced.

Since both GEC and EPS have closed pores, and the aerogel used in this study is hydrophobic, the degradation of these renders (B(GEC), C(SA) and D(EPS)) only occurs at the aggregate/matrix interface or in the renders matrix.

Regarding the compressive strength, an increase (between 1% and 19 %, Table 3) is recorded in lightweight renders at the end of the freeze/thaw cycles, which is contradictory to the weight loss. However, this behaviour can be explained by an extra curing effect caused by freezing/thawing cycles due to the Na^+^ [59,60] that compensate to some extent the external degradation. These results meet the study of Zhang & Gjorv [61] (as quoted by [62]) that obtained equal or even better durability for lightweight aggregate concrete than standard concrete, even when subjected to severe conditions.

**Figure 3:**
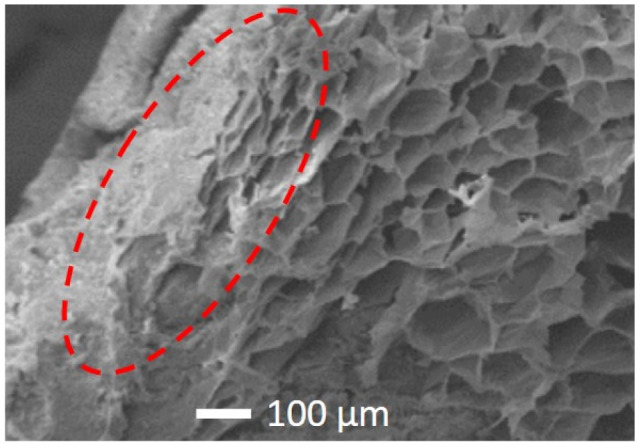
SEM image of D(EPS) render.

The high improvement in the compressive strength of EPS-based render (D(EPS)) and the small variation in its weight (Table 3) can be explained by the fact that EPS can absorb part of the freeze crystallization pressure, reducing render deterioration [63]. As shown in Figure 3 with scanning electron microscopy (SEM), a deformation of the EPS border cells with the cement paste is visible.

Concerning the elasticity modulus behaviour, Table 3, the renders C(SA) and B(GEC) show the smallest variation of Young’s modulus. In turn, the render D(EPS) varies by 42% after freeze/thaw cycles, which is too high according to ASTM C 666 [64], that states that materials must retain at least 60% of initial Young’s modulus after 300 freeze/thaw cycles. Although this criterion is for application to concrete structures, it provides an idea of renders behaviour to freeze/thaw cycles.

As regards thermal behaviour, render C(SA) had the best performance with a reduction in thermal conductivity, while the remaining renders maintained the values registered before the freeze/thaw cycles (Table 3). However, the reduction of the thermal conductivity in the render C(SA) may be related to the less flat surface of these renders after freeze/thaw cycles. The roughness of the render surface creates small air pockets between the probe of the measuring equipment and the surface of the test specimen, with probable resulting in the reduction in the value obtained in the thermal conductivity test [65].

In general, the expanded cork-based render (B(GEC)) presented the best behaviour to the freeze/thaw cycles since the aerogel-based render (C(AS)) showed a marked reduction of weight and the EPS-based render (D(EPS)) showed a substantial reduction in Young’s modulus. One possible explanation is the higher specific heat capacity of cork (1560 J/kg K) compared to EPS (1430 J/kg K) and especially to aerogel (740 J/kg K), which makes the behaviour of cork less susceptible to temperature variations [19,25,66].

These results agree with the study of Oliveira & Abidi [48], where, when comparing cork-based renders and EPS-based renders, registered a smaller reduction of the dynamic elasticity modulus in cork-based renders.

### 2.2. Hygrothermal and IR Accelerated Aging

To evaluate the effects of accelerated aging with hygrothermal and infrared radiation cycles, the specimens were weighed and tested for compressive strength, ultra-sound velocity, and thermal conductivity before and after accelerated aging cycles, with the results summarized in Table 4.

**Figure 4:**
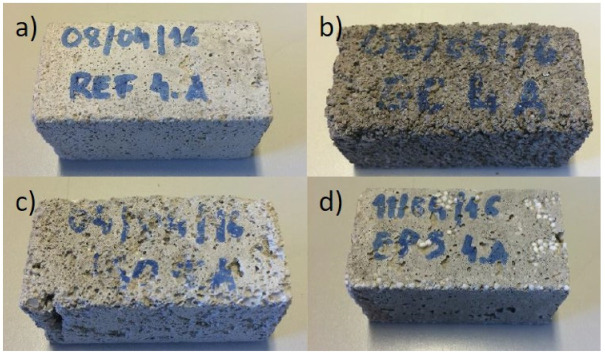
Half standard specimens after hygrothermal and IR accelerated aging cycles: (**a**) A(SS); (**b**) B(GEC); (**c**) C(SA); (**d**) D(EPS).

In Figure 4, it is possible to visualize the degradation caused by hygrothermal and IR accelerated aging cycles of the surface layer of renders B(GEC), C(AS), and D(EPS), caused by their leaching, but in none of the used specimens for the evaluation of mass variation and compressive strength were cracking or loss of section observed.

**Figure 5:**
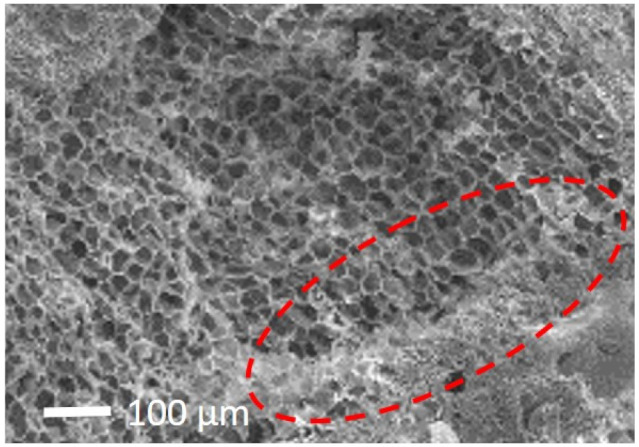
SEM image of B(GEC) render.

When analysing the results obtained before and after these accelerated aging cycles, a significant increase of weight in render B(GEC) stands out when compared to the other renders. A justification may lie in the high compressibility of cork [67,68] and a larger space in the border cells (open cells) [69], as shown in Figure 5, which allows a more extended hydration reaction due to temperature along the accelerated aging [45], that can lead to a reduction of porosity [70].

It was expected a higher increase of compressive strength in GEC-based render than in EPS-based render, which although the latter has also a high compressibility [71], offers less space at the outer boundary for the formation of hydration reaction compounds [45,72,73]. However, damage on the walls of outer cells of GEC may occur [52], as in EPS may occur improvements in the hydrated cement paste-EPS interface which allows more adherence between both [63,73]. This may result in a stronger aggregate/paste bond in the EPS-based render than the resultant in the GEC-based render.

**Figure 6:**
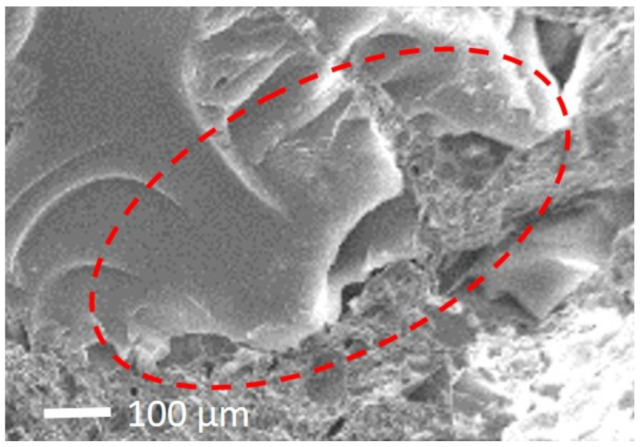
SEM image of C(SA) render.

In its turn, the aerogel has a reduced compressibility (very close to that of bulk vitreous silica) [74], which may explain the smallest increase in mass among lightweight renders. Since the silica aerogel has pores with diameter less than 0.1 µm [75], smaller than the size of the resulting compounds of the cement hydration reactions (higher than 0.5 µm) [76], its surface acts as a flat surface (Figure 6), leaving no room for the formation of hydration compounds in the outer pores, leading to the smallest increase of mechanical strength between light renders. Still, all lightweight renders kept the class of strength CSI (and render D(EPS) may be included in class CSII), as before the accelerated aging test.

**Figure 7:**
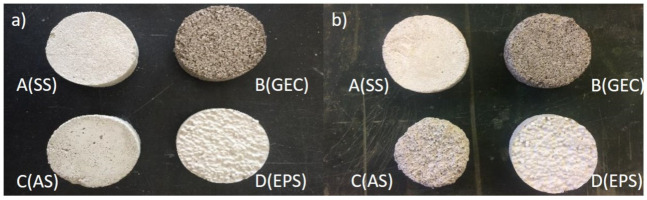
Specimens for thermal conductivity evaluation after hygrothermal and IR accelerated aging cycles: (**a**) before; (**b**) after.

As regards ultra-sound velocity, Table 4, all variations were less than 10%, therefore no significant variations were considered since it falls within the variability range of the test. In the analysis of the evolution of the thermal behaviour with the accelerated aging, apart from the control render A(SS) that showed a significant increase (from 1.18 to 2.12 W/m.K), renders with lightweight aggregates did not undergo significant changes, with B(GEC) and D(EPS) render showing an increase of 0.02 W/m.K and no changes at C(SA) render. However, the value of C(SA) render may not correspond to reality once it occurred loss of specimen volume with the accelerated aging cycles, as can be seen in Figure 7, resulting in a rough surface that creates air pockets that interfere with the measurement of thermal conductivity through the flat probe of ISOMET 2116 equipment.

Combining the results of hygrothermal and IR accelerated aging (AA) cycles and freeze/thaw (F/T) cycles (Table 5) it is possible to see that, in general, AA cycles cause an increase in mortar mass, more pronounced in renders with lightweight aggregates, while F/T cycles cause a reduction in mass, with greater significance in mortar C(SA). These variations were not equally reflected in the compressive strength, which showed an increase, regardless of the increase or decrease in mass, except for the F/T cycle in mortar A(SS) which caused a decrease in compressive strength. In fact, it is possible to see that, among renders with lightweight aggregates, B(GEC) and D (EPS) renders are those that have better mechanical strength after AA and F/T cycles, but that present worse performance in thermal behaviour when submitted to AA cycles. Regarding the ultra-sound velocity, although a visible trend towards an increase in the values of mortars with light aggregates after the AA cycles and a reduction after the F/T cycles, the variation was not sufficient to be considered significant.

## 3. Conclusions

In this study, the response of thermal insulation renders to degradation actions was evaluated and discussed, namely freeze/thaw and hygrothermal and IR cycles. Different trends in weight variation were registered. An increase in weight was recorded with accelerated aging hygrothermal and IR cycles (between 4% and 29 %). With the freeze/thaw cycles there was a reduction in weight (between 3% and 15 %), which is indicative of renders degradation.

Nevertheless, with the freeze/thaw cycles a greater increase in compressive strength was observed in renders with regranulated cork (GEC) and expanded polystyrene (EPS), 14.29% and 19.07% respectively, than with hygrothermal and IR accelerated aging cycles for the same renders (6.78% and 10.90 % respectively).

Regarding the thermal behaviour, apart from render with silica aerogel, C(SA), there was a considerable increase in the thermal conductivity value of renders after hygrothermal and IR cycles, while freeze/thaw cycles had no influence on this property. Despite the weight loss there was a slight increase in compressive strength after both accelerated aging cycles.

Thus, silica aerogel-based render with a thermal conductivity between 0.08 and 0.07 W/m.K (lowest value of the studied renders) and a mass reduction of 14.52% after the freeze/thaw cycles indicate that this render is suitable for a heat-insulating render, but further study of its formulation and interaction with the interface aggregates and binder matrix is necessary.

Moreover, the EPS-based render, with a considerable reduction of the Young’s modulus, has shown an inappropriate behaviour after aging cycles. On the other hand, the GEC-based render showed lower susceptibility to the degradation phenomena in this study, presenting only greater variation in thermal conductivity value with the accelerated aging cycles.

In the course of this work, we also note the lack of normative information regarding thermal render aging cycles when compared to standards and procedures that already exist for the accelerated aging of concrete.

This study highlights the importance of developing studies of durability of thermal renders and the important contribution of each compound (aggregates, binder, admixtures) on durability performance. Further studies should focus different formulations, especially in the case of aerogel-based renders. Furthermore, the interface lightweight aggregate/matrix as well as the binder matrix self-behaviour should be deeply studied as well as the effects after different types of accelerated degradation mechanisms to characterize better in-service performance of thermal renders.

## 4. Materials and Methods

**Figure 8:**
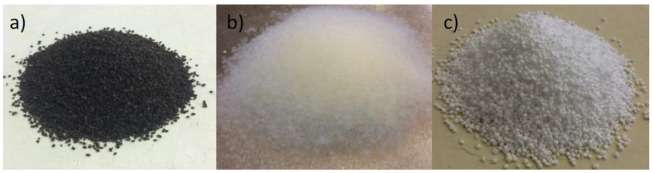
Lightweight aggregates used in the study: (**a**) regranulated expanded cork (GEC); (**b**) hydrophobic silica aerogel (SA); and (**c**) expanded polystyrene (EPS).

For evaluation of durability of thermal renders, renders with silica aerogel—SA—(0.018 to 0.020 W/m.K), regranulated expanded cork—GEC—(0.03948 W/m.K) and expanded polystyrene—EPS—(0.032 to 0.040 W/m.K) (with bulk densities of 66.9; 51.9 and 11.0 kg/m^3^, respectively) (Figure 8) were produced. As a control formulation, one render with silica sand was also produced (bulk density of 1224.6 kg/m^3^). The renders were produced in a volumetric ratio 1:4 (binder:aggregate), with Portland cement CEM I B-L 32.5 N as a binder.

To obtain a good mixture with the silica aerogel used in this study, the addition of the following admixtures was required: surfactant, cellulose ether and resin, in proportions of 0.5 wt.%, 0.075 wt.% and 2 wt.% of binder, respectively [51]. The content of these admixtures was the same in the renders with lightweight aggregates, resulting in the mixes presented in Table 6.

**Figure 9:**
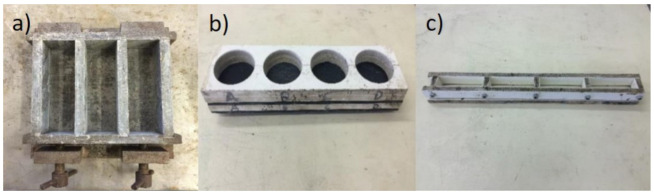
Moulds used in production of specimens: (**a**) standard specimens (40 × 40 × 160 mm^3^); (**b**) thermal conductivity specimens (60 mm diameter and 20 mm thickness); (**c**) small-size specimens (20 × 20 × 80 × mm^3^).

The renders were mixed and cured by 28 days in accordance to EN 1015-11 [77]. Six standard specimens (40 × 40 × 160 mm^3^) were moulded. Two small-size specimens (cylindrical, with 60 mm diameter and 20 mm thickness) were also produced for thermal conductivity tests and one small-size specimens (20 × 20 × 80 × mm^3^) for Young’s modulus test, using the moulds in Figure 9, resulting in the measurements displayed in Table 7. For the control of renders at fresh state, the determination of consistence was performed by flow table according to EN 1015-3 [78].

**Figure 10:**
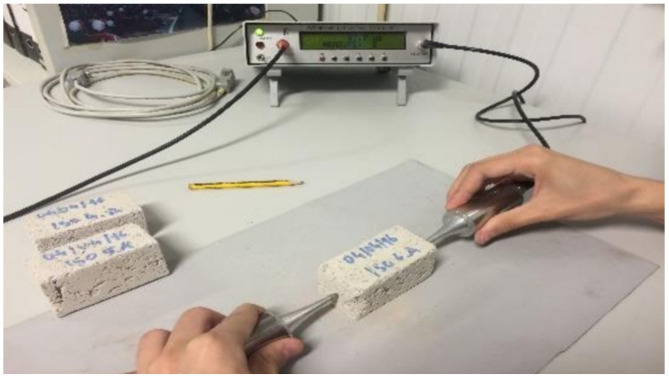
Ultra-sound velocity test apparatus.

The pulse ultra-sound velocity was carried out by direct method according to Fe Pa 43. [79], as shown in Figure 10, with Steinkamp ultrasonic tester BP-7 equipment with exponential transducers of 50 kHz. The compressive strength test was conducted according to EN 1015-11 [77] with, Form Test—Seider, model D-7940 equipment. The Young’s modulus was determined according to ASTM E 1876-1 [80], through the resonant frequency of the flexural vibration mode obtained with the equipment GrindoSonic MK5 “Industrial”. The thermal conductivity was evaluated by the transient method with the ISOMET 2114 device, in line with ASTM D 5930 [81], and was considered as suitable method for testing in small scale samples [62]. These properties were evaluated before and after aging tests.

To visualize the interaction between aggregate border and cement paste, thermal renders were observed by scanning electron microscopy (SEM) JEOL JSM-7001F equipment, at 28 days age.

The freeze/thaw aging tests were carried out in Laboratory of Construction, of Instituto Superior Técnico (IST), University of Lisbon. The hygrothermal and infrared radiation aging test were performed in the National Laboratory of Civil Engineering (LNEC).

### 4.1. Accelerated Aging—Freeze/Thaw Cycles

**Figure 11:**
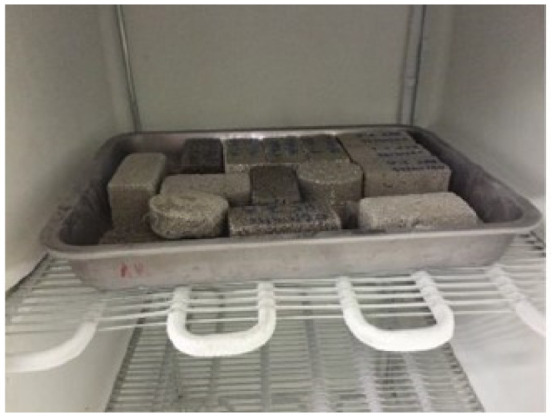
Freezer at −15 ± 1 °C.

**Figure 12:**
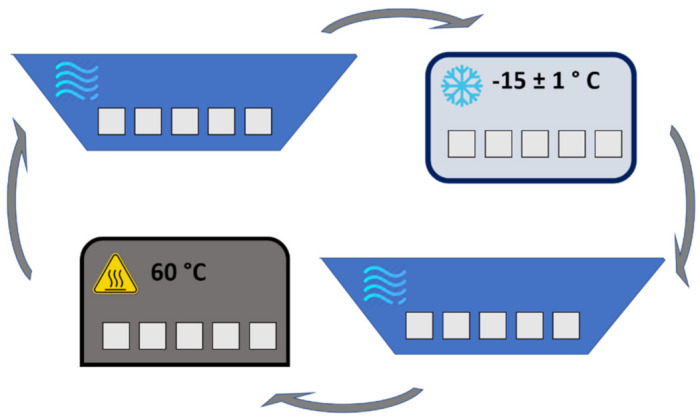
Schematic representation of a Freeze/Thaw cycle.

In order to evaluate the degradation of renders due to freeze/thaw cycles, the procedure was adapted from EN 1348 [82]. The equipment required consisted of: scale with 0.01g of resolution, oven at 60 ± 2 °C; freezer at −15 ± 1 °C (Figure 11) and a water container at 20 ± 2 °C and it was performed with the following steps, as schematically shown in Figure 12:

- Setting of the specimens immersed in water container for at least 12 h;
- Setting of the specimens in a freezer at -15 ± 1 °C during 4 h;
- Setting of the specimens immersed in water container at 20 ± 2 °C during 4 h;
- Setting of the specimens in an oven at 60 °C.

The cycle described above is repeated until a mass variation of more than 30% was obtained, or at a maximum of 20 cycles [83].

**Figure 13:**
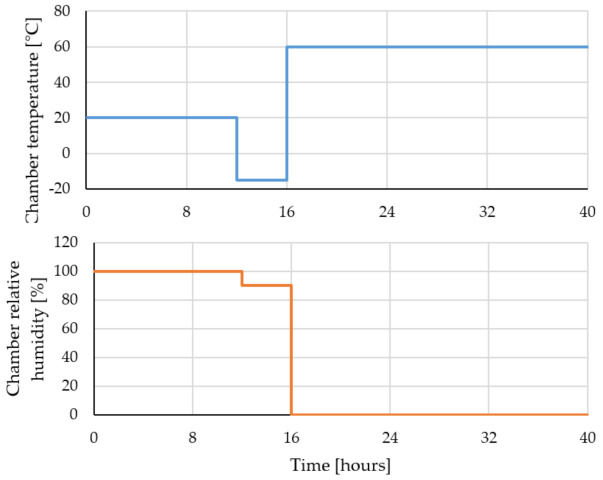
One freeze/thaw cycle.

Figure 13 shows a chart with the temperature and air relative humidity variation of the chambers in which the specimens were subjected during one cycle of freeze/thaw.

### 4.2. Accelerated Aging—Hygrothermal and Infrared Radiation (IR) Cycles

**Figure 14:**
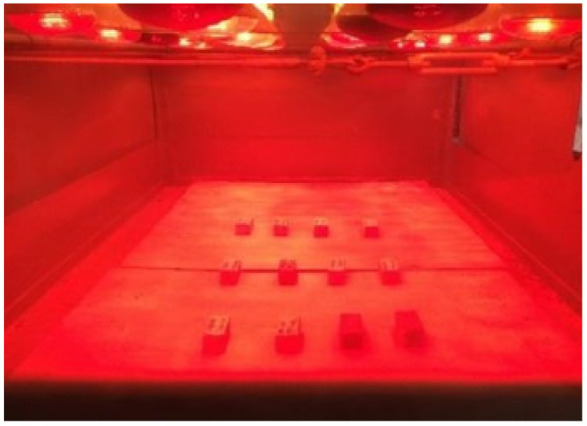
Infrared aging chamber at 60 ± 2 °C.

In order to evaluate the durability of the renders to the degrading agents, the specimens were subjected to accelerated aging through combined actions of freeze, rain and heat through infrared radiation. This procedure is very important to evaluate the degradation of renders under climatic agent exposure [15]. The accelerated aging procedure is intended to reproduce the effects of normal aging of render specimens through accelerated aging cycles. The equipment required consisted of: an infrared aging chamber at 60 ± 2 °C with water sprinklers (Figure 14); a freezer at −15 ± 1 °C and a climatic chamber at 20 ± 2 °C and RH of 65 ± 5%. The test was performed based on the methodology of EN 1015-21 [84], with the following steps:

(a) Eight heat-freeze cycles, where each cycle consists of:
  - infrared heating of specimens, at 60 ± 2 °C during 8 h ± 15 min;
  - setting of the specimens in a climate chamber at 20 ± 2 °C and RH of 65 ± 5 % during 30 min ± 2 min;
  - cooling in a freezer at −15 ± 1 °C during 15 h ± 15 min;
  - setting of the specimens in a climate chamber at 20 ± 2 °C and RH of 65 ± 5 % during 30 min ± 2 min.
(b) Before the freeze-thaw cycle begins and after the end of the freeze-heat cycle, the specimens are subjected to standard conditions of 20 ± 2 °C and RH of 65 ± 5 % at least for 48 h.
(c) Eight freeze-thaw cycles, where each cycle consists of:
  - sprinkling the specimens with water at 20 ± 2 °C during 8 h ± 15 min;
  - setting of the specimens in a climate chamber at 20 ± 2 °C and RH of 65 ± 5 % during 30 min ± 2 min;
  - cooling in a freezer at −15 ± 1 °C during 15 h ± 15 min;
  - setting of the specimens in a climate chamber at 20 ± 2 °C and RH of 65 ± 5 % during 30 min ± 2 min.
(d) After the end of the freeze-thaw, the specimens are subjected to standard conditions of 20 ± 2 °C and RH of 65 ± 5 % at least for 48 h.

**Figure 15:**
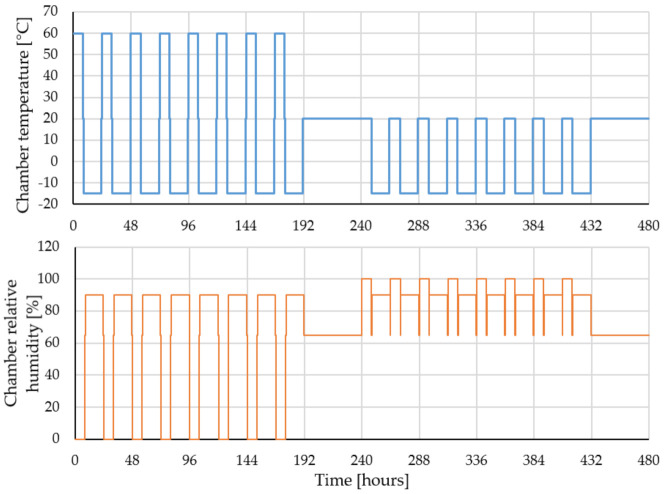
Total accelerated hygrothermal aging cycles.

Figure 15 illustrates the temperature and relative humidity variation of the chambers in which the specimens were subjected during the cycles of accelerated aging procedure.

## Figures and Tables

**Table 1:** Properties of renders at fresh state.

Renders	Bulk Density of Fresh Render [kg/m^3^]	Flow Value [mm]
A(SS)	1893	181
B(GEC)	508	147
C(SA)	607	135
D(EPS)	432	165

**Table 2:** Dry bulk density of the renders at hardened state.

Renders	A(SS)	B(GEC)	C(SA)	D(EPS)
Dry bulk density [kg/m^3^]	1823	443	438	458

**Table 3:** Results from freeze/thaw cycles.

Renders	Weight [g]			Cs [N/mm^2^]			USV [m/s]			E-mod [N/mm^2^]			λ [W/m.K]		
	B_F/T	A_F/T	Var	B_F/T	A_F/T	Var	B_F/T	A_F/T	Var	B_F/T	A_F/T	Var	B_F/T	A_F/T	Var
A(SS)	227.19	220.24	−3.06	19.42	18.96	−2.37	1909	1879	−1.57	13,536	11,884	−12.20	1.51	1.51	0.00
B(GEC)	58.72	54.75	−6.76	1.26	1.44	14.29	870	840	−3.45	304	322	5.92	0.08	0.08	0.00
C(SA)	55.52	47.46	−14.52	0.72	0.73	1.39	890	850	−4.49	449	496	10.47	0.08	0.07	−12.50
D(EPS)	60.05	56.97	−5.13	2.15	2.56	19.07	1220	1132	−7.21	1344	769	−42.78	0.1	0.1	0.00

B_F/T—before freeze/thaw; A_F/T—after freeze/thaw; Var—variation in % from B_F/T to A_F/T; Cs—compressive strength; USV—ultra-sound velocity; E-mod—Young’s modulus; λ—thermal conductivity.

**Table 4:** Results from hygrothermal and IR accelerated agingf cycles.

Renders	Weight [g]			Cs [N/mm^2^]			USV [m/s]			λ [W/m.K]		
	B_AA	A_AA	Var	B_AA	A_AA	Var	B_AA	A_AA	Var	B_AA	A_AA	Var
A(SS)	232.46	243.07	4.56	17.81	14.86	-16.56	1986	1951	−1.76	1.18	2.12	79.66
B(GEC)	56.89	73.26	28.77	1.18	1.26	6.78	915	934	2.08	0.08	0.10	25.00
C(SA)	57.13	64.66	13.18	0.83	0.85	2.41	908	974	7.27	0.07	0.07	0.00
D(EPS)	59.87	68.91	15.10	2.11	2.34	10.90	1192	1249	4.78	0.11	0.13	18.18

B_AA—before accelerated aging; A_AA—after accelerated aging; Var—variation in % from B_AA to A_AA; Cs—compressive strength; USV—ultra-sound velocity; λ—thermal conductivity.

**Table 5:** Variation in % after accelerated aging cycles.

Renders	Weight		Compressive Strenght		Ultra-Sound Velocity		Young’s Modulus		Thermal Conductivity	
	AA	F/T	AA	F/T	AA	F/T	AA	F/T	AA	F/T
A(SS)	4.56	−3.06	16.56	−2.37	−1.76	−1.57	n.t.	−12.20	79.66	0.00
B(GEC)	28.77	−6.76	6.78	14.29	2.08	−3.45	n.t.	5.92	25.00	0.00
C(SA)	13.18	−14.52	2.41	1.39	7.27	−4.49	n.t.	10.47	0.00	−12.50
D(EPS)	15.10	−5.13	10.90	19.07	4.78	−7.21	n.t.	−42.78	18.18	0.00

AA—after accelerated aging; F/T—after freeze/thaw; Green color cells—improvement over 10%; Red color cells—deterioration over 10%; Without color (with cells)—light variation: n.t.—not tested.

**Table 6:** Identification of the render samples according to lightweight aggregates.

Renders		A(SS)	B(GEC)	C(SA)	D(EPS)
Water:binder ratio (wt)		1.00	0.79	0.63	0.62
Aggregate (vol.%)	Silica sand (≤ 2 mm)	100	-	-	-
	Regranulated expanded cork (0.5–2 mm)	-	100	-	-
	Silica aerogel (≤ 2 mm)	-	-	100	-
	Expanded polystyrene (2–4 mm)	-	-	-	100
Admixture (wt% relative to total binder)	Surfactant	-	0.5	0.5	0.5
	Cellulose ether	-	0.075	0.075	0.075
	Resin	-	2	2	2

**Table 7:** Identification of the render samples according to lightweight aggregates.

Degradation Procedures	Test	Specimen	Number of Measurements
Freeze/Thaw	Cs	Standard	6
	USV	Standard	180
	E-mod	20 × 20 × 80 × mm^3^	20
	λ	Cylindrical	2
Accelerated aging	Cs	Standard	6
	USV	Standard	180
	λ	Cylindrical	2

Cs—compressive strength; USV—ultra-sound velocity; E-mod—Young´s modulus; λ—thermal conductivity.

## Data Availability

The data was produced in the Master Thesis of the first Author.
